# Comparison of Sex Differences in Outcomes of Patients With Aneurysmal Subarachnoid Hemorrhage: A Single-Center Retrospective Study

**DOI:** 10.3389/fneur.2022.853513

**Published:** 2022-04-28

**Authors:** Yuankun Cai, Zheng Liu, Chenguang Jia, Jingwei Zhao, Songshan Chai, Zhengwei Li, Chengshi Xu, Tingbao Zhang, Yihui Ma, Chao Ma, Xinjun Chen, Pucha Jiang, Wenyuan Zhao, Jincao Chen, Nanxiang Xiong

**Affiliations:** Department of Neurosurgery, Zhongnan Hospital of Wuhan University, Wuhan, China

**Keywords:** aneurysmal subarachnoid hemorrhage (aSAH), gender, propensity adjustment, outcome, aneurysm

## Abstract

**Background:**

Sex differences in the outcomes of patients with aneurysmal subarachnoid hemorrhage (aSAH) remain controversial. The aim of this study was to evaluate sex differences in the outcomes of patients with aSAH.

**Method:**

This study retrospectively analyzed the clinical data of consecutive patients with aSAH, admitted to the Department of Neurosurgery, Wuhan University Zhongnan Hospital, from May 1, 2020 to December 31, 2020. The modified Rankin Scale (mRS) score was used to evaluate the prognosis of patients at discharge. Outcome indicators included cerebral ischemia, hydrocephalus, and mRS ≥ 2 at discharge.

**Results:**

The majority (65%) of the 287 patients with aSAH included in the study were females. Patients were divided into female (*n* = 184) and male (*n* = 99) groups; the female patients were significantly older than the male patients (61.3 ± 8.5 years vs. 60.0 ± 8.5 years, *p* = 0.032). The incidence of comorbidities (hypertension, diabetes, and heart disease) was higher in the female group than in the male group, but the difference was not statistically significant. Although more female patients than male patients underwent endovascular treatment, there was no statistical difference in the treatment approach between the two groups. Comparison of post-operative complications and mRS scores at discharge revealed that the rate of cerebral ischemia and mRS ≥ 2 at discharge were significantly higher among female patients than among male patients. Moreover, this difference persisted after propensity adjustment for age and treatment approach. Analysis of risk factors for poor prognosis at discharge in both pre- and post-adjustment patients revealed cerebral ischemia and high mFisher score (mFisher = 3/4) to be independent risk factors.

**Conclusion:**

Female patients with aSAH have a worse prognosis than male patients, and this difference may be because women are more susceptible to cerebral ischemia.

## Introduction

Stroke is the second leading cause of mortality and disability-adjusted life-years lost worldwide, affecting almost 14 million individuals annually (1). Epidemiological studies have found that there are sex differences in many aspects of stroke, such as female patients having different vascular risk factors (2) and a greater likelihood of a poor prognosis (3) when compared with male patients. Aneurysmal subarachnoid hemorrhage (aSAH) is a common type of hemorrhagic stroke with a mortality rate of up to 30%, and a higher mortality rate in survivors even after successful treatment when compared to the general population (4). A previous study showed that female patients were more likely to develop intracranial aneurysms, especially seen among elderly patients (5). Furthermore, women also have a higher incidence and mortality rate of aSAH (6, 7). However, sex differences in aSAH outcomes remain controversial. The main reason for the different conclusions may be the different basic characteristics of male and female patients and the large number of confounding factors (8). Female patients, on average, are older than male patients and have more comorbidities. Moreover, different treatment approaches may also have different effects on patient outcomes (9).

Therefore, this study retrospectively analyzed the clinical data of consecutive patients with aSAH treated in our institution. Propensity scores were used to adjust for age and treatment approach between male and female patients. We compared the differences in outcomes between the two groups before and after adjustment.

## Method

### Material Collection and Variables

Institutional Review Board/Ethics Committee approval was not required for this retrospective analysis of de-identified Medicare data. Likewise, patient consent was not necessary in our study. Patients diagnosed with aSAH from May 1, 2020 to December 31, 2020, in the Department of Neurosurgery, Wuhan University Zhongnan Hospital, were collected. To ensure the accuracy of the analysis, the patients who were considered to have aSAH on admission but had no definite aneurysm on digital subtraction angiography (DSA) were excluded. Patients younger than 18 years of age were also excluded. Patients who had already undergone aneurysm surgery at other hospitals and were transferred to our hospital for further treatment were excluded.

Patients' basic characteristics, comorbidities, smoking and drinking status, aneurysm characteristics, treatment approach, and inpatient logs were reviewed in detail. The location and number of aneurysms were clarified by reviewing the patient's computed tomography angiography (CTA) or DSA. If the patient has multiple intracranial aneurysms, the one that ruptured is recorded as the responsible aneurysm. Aneurysms originating from all segments of the vertebral artery and basilar artery and their branch aneurysms were recorded as posterior circulation artery (PCA) aneurysms. The treatment of aneurysm is divided into craniotomy, interventional treatment, and conservative treatment. Various endovascular treatment approaches, such as coiling and flow direction, are collectively referred to as interventional treatments. Very few patients treated with a combination of clipping and intervention or bypass were classified as receiving craniotomy. Those who underwent only extraventricular drainage and/or decompressive craniectomy were classified as having received conservative treatment.

### Management and Assessment

Cranial computed tomography (CT) and CTA were performed when a patient was considered to have aSAH in the emergency department. If there was no clear diagnosis or characterization of aneurysm, further DSA was performed. All patients diagnosed with aSAH in our department were treated with nimodipine, an anti-vascular spasm agent, after admission. Aneurysm management was performed by two chief surgeons specializing in craniotomy and intervention, respectively. Patient admission and imaging findings were reviewed to obtain the Hunt–Hess grade, Glasgow Coma Scale (GCS), and modified Fisher score. The presence of cerebral ischemia and hydrocephalus was assessed using the patient's pre-discharge cranial CT imaging and clinical manifestations. The patient's clinical symptoms, antibiotic use, and cerebrospinal fluid examination results were used to determine whether the patient had complications of intracranial infection.

Inpatient and discharge data and patient discharge condition assessed using the modified Rankin Scale (mRS) score were reviewed. An mRS of 2 represents mild disability, an inability to perform all tasks and activities, yet able to live normally without depending on others. Therefore, we chose the more stringent criterion of mRS 0–1 as an indicator of prognostic excellence. Patients with an mRS ≥2 were defined as having a poor outcome. The above scoring and assessments were performed independently by two individuals and reviewed and finalized by the supervising physician.

### Statistics Analysis

Statistical analyses were performed using SPSS version 26 (IBM Corp., Armonk, NY, USA). Patient age and body mass index (BMI) are continuous variables described as mean (±SD) or median (interquartile range), while comorbidities, smoking and drinking status, aneurysm characteristics, and treatment approach and complications are count variables and represented as rates. Differences between sexes were assessed using chi-squared analysis, Fisher's exact test, Student's *t*-test, and the Kruskal–Wallis one-way analysis of variance, as appropriate. A value of *p* ≤ 0.05 was considered statistically significant. Patients were divided into age classes at 10-year intervals, and propensity matching was performed for male and female groups according to both “age class” and “treatment approach.” Univariate analysis of poor prognosis was first performed, and the results were then included in a multivariate logistic regression analysis system to analyze the independent risk factors for poor patient prognosis. Microsoft Excel software was used for graphing.

## Results

### Patient Characteristics

**Figure 1:**
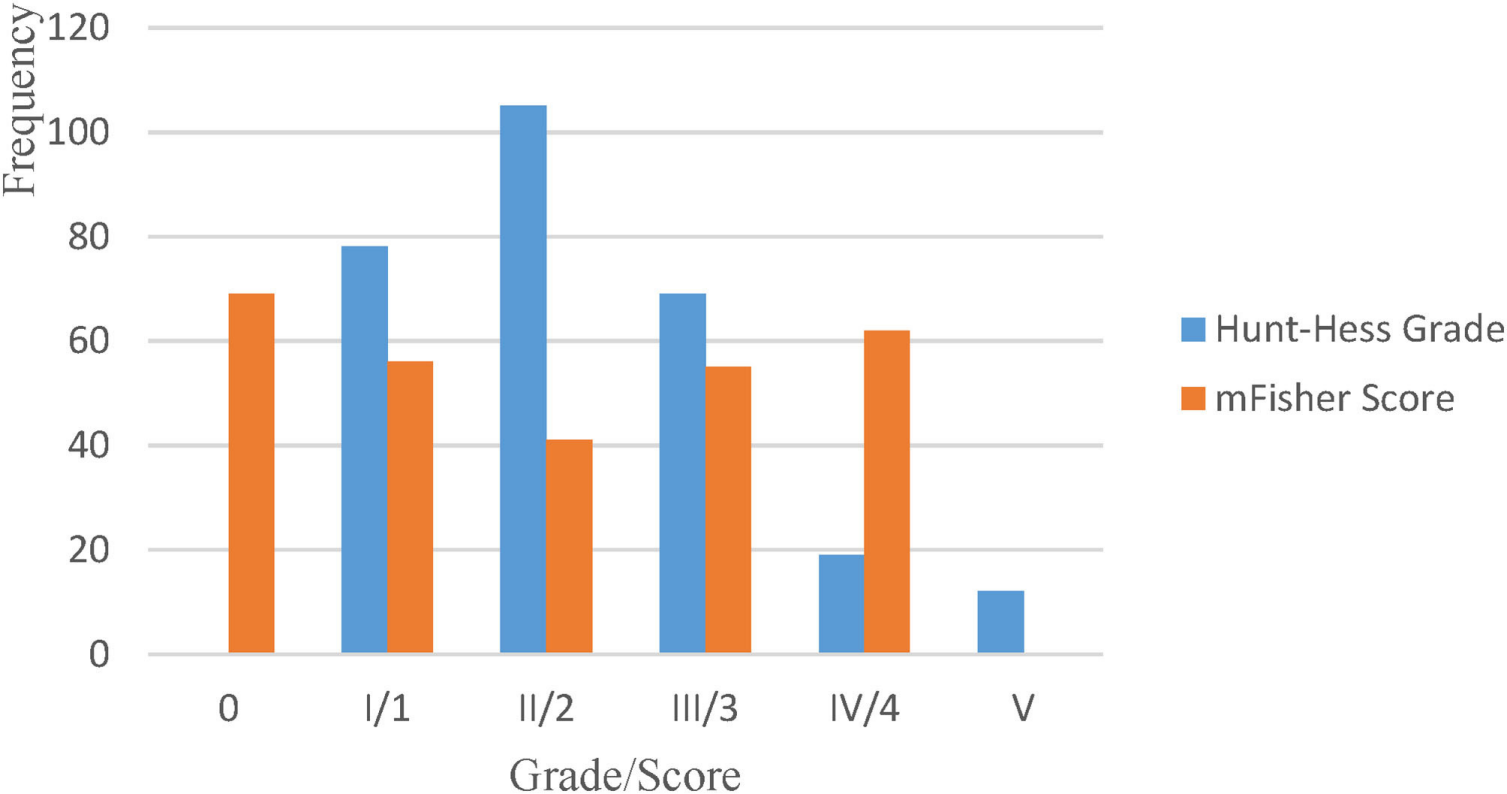
The overall distribution of patients' Hunt–Hess grades and mFisher scores.

A total of 287 patients with a diagnosis of aSAH were reviewed in this study; four patients were excluded because no definite aneurysm was found on either CTA or DSA after admission. A predominantly female population (65.0%) of 283 patients with a mean age of 60.5 ± 8.6 years (range, 34–85 years) was included in the analysis (Table 1). Approximately half of the patients were overweight or obese (BMI ≥ 24 kg/m^2^), and more than half had comorbid hypertension. Approximately a quarter of the patients were smokers or drinkers, the majority of whom were men. Aneurysms mainly originated from arteries in the anterior circulation (87.3%) and were found most commonly in the internal carotid artery (ICA) (51.6%), followed by the anterior cerebral artery (ACA) (19.8%). Multiple aneurysms were present in approximately one fifth of the patients (*n* = 49, 19.8%). A clear majority of patients underwent aggressive surgical treatment; 60% underwent craniotomy and 31% underwent interventional treatment. The Hunt–Hess grades of patients were predominantly low grade (I and II), with a high grade (III–V) present in ~30% of the patients, and the mFisher score was almost equally divided in each score (Figure 1).

**Table 1:** The overall characteristics of the patients and the comparison of gender differences.

	**Total (*n* = 283)**	**Female (*n* = 184)**	**Male (*n* = 99)**	***P***
**Age (mean ± SD)**	60.5 ± 8.6	61.3 ± 8.5	60.0 ± 8.5	0.032^*^
**BMI (mean ± SD)**	23.8 ± 3.4	23.9 ± 3.6	23.6 ± 3.0	0.505
<18.5	14 (4.9%)	11 (6.0%)	3 (3.0%)	0.044^*^
18.5 ~ 23.9	131 (46.3%)	80 (43.5%)	51 (51.5%)	
24 ~ 27.9	109 (38.5%)	68 (37.0%)	41 (41.4%)	
≥28	29 (10.2%)	25 (13.6%)	4 (4.0%)	
**Comorbidities**				
Hypertension	162 (57.2%)	110 (59.8%)	52 (52.5%)	0.239
Diabetes	18 (6.4%)	15 (8.2%)	3 (3.0%)	0.092
Heart disease	22 (7.8%)	17 (9.2%)	5 (5.1%)	0.209
Smoking	73 (25.8%)	7 (3.8%)	66 (66.7%)	<0.001^*^
Drinking	60 (21.2%)	12 (6.5%)	48 (48.5%)	<0.001^*^
Multiple aneurysms	49 (17.3%)	35 (19.0%)	14 (14.1%)	0.301
**Aneurysm location**				<0.001^*^
ACA	56 (19.8%)	22 (12.0%)	34 (34.3%)	
ICA	146 (51.6%)	111 (60.3%)	35 (35.4%)	
MCA	45 (15.9%)	28 (15.2%)	17 (17.2%)	
PCA	36 (12.7%)	23 (12.5%)	13 (13.1%)	
**Treatment**				0.29
Craniotomy	170 (60.1%)	106 (57.6%)	64 (64.6%)	
Endovascular	88 (31.1%)	63 (34.2%)	25 (25.3%)	
Conservative	25 (8.8%)	15 (8.2%)	10 (10.1%)	

*BMI, Body mass index; ICA, Internal carotid arteries; ACA, Anterior cerebral artery; MCA, Middle cerebral artery; PCA, Posterior circulation artery*.

* *p < 0.05, with a statistical difference*.

### Sex Differences and Adjustment

The patients were divided into a female group (*n* = 184) and male group (*n* = 99) according to their sex, with a ratio of ~2:1 in group size (Table 1). Female patients were significantly older than male patients (*p* = 0.032) and a greater proportion was obese (13.6 vs. 4.0%). Although female patients had higher rates of comorbidities, this difference was not statistically significant. The proportion of smokers and drinkers was significantly higher in male patients than in female patients (*p* < 0.001). There was no significant difference in the proportion of patients with multiple aneurysms between the two groups; however, there was a significant difference in the origin of aneurysms. Most female patients (60.3%) had aneurysms originating from the ICA, whereas male patients had a similar proportion of aneurysms originating from the ICA and the ACA. Both groups were mainly treated with craniotomy. However, the proportion of patients who received interventional treatment was higher in the female group than in the male group (34.2 vs. 25.3%).

The difference in mFisher scores between the two groups was not significant and was evenly distributed between scores (Table 2, Figure 1). The comparison of complications showed that female patients were significantly more likely to have cerebral ischemia than male patients (27.2 vs. 15.2%, *p* = 0.022). There was no significant difference between the two groups regarding the incidence of mortality, hydrocephalus, or intracranial infection. The proportion of female patients with an mRS score ≥2 was significantly higher than that of male patients at discharge (*p* = 0.013).

**Table 2:** Comparison of differences in outcomes between male and female patients before and after propensity adjustment.

	**Before the adjustment**		***P***	**After the adjustment**		***P***
	**Female (*n* = 184)**	**Male (*n* = 99)**		**Female (*n* = 168)**	**Male (*n* = 63)**	
**mFisher**						
0	50 (27.2%)	19 (19.2%)	0.225	43 (25.9%)	19 (22.9%)	0.68
1	37 (20.1%)	19 (19.2%)		36 (21.7%)	15 (18.1%)	
2	22 (12.0%)	19 (19%2)		19 (11.5%)	14 (16.9%)	
3	32 (17.4%)	23 (23.2%)		30 (18.1%)	18 (21.7%)	
4	43 (23.4%)	19 (19.2%)		38 (22.9%)	17 (20.5%)	
**Complications**						
Mortality	18 (9.8%)	11 (11.1%)	0.725	16 (9.6%)	10 (12.0%)	0.558
Ischemia	50 (27.2%)	15 (15.2%)	0.022^*^	47 (28.3%)	14 (16.9%)	0.048^*^
Hydrocephalus	25 (13.6%)	12 (12.1%)	0.727	24 (14.5%)	9 (10.8%)	0.428
Intracranial infection	1 (0.5%)	2 (2.0%)	0.247	1 (0.6%)	2 (2.4%)	0.218
mRS ≥ 2	110 (59.8%)	44 (44.4%)	0.013^*^	100 (60.241)	39 (46.988)	0.047^*^

*mRS, Modified rankin scale*.

* *p < 0.05, with a statistical difference*.

**Figure 2:**
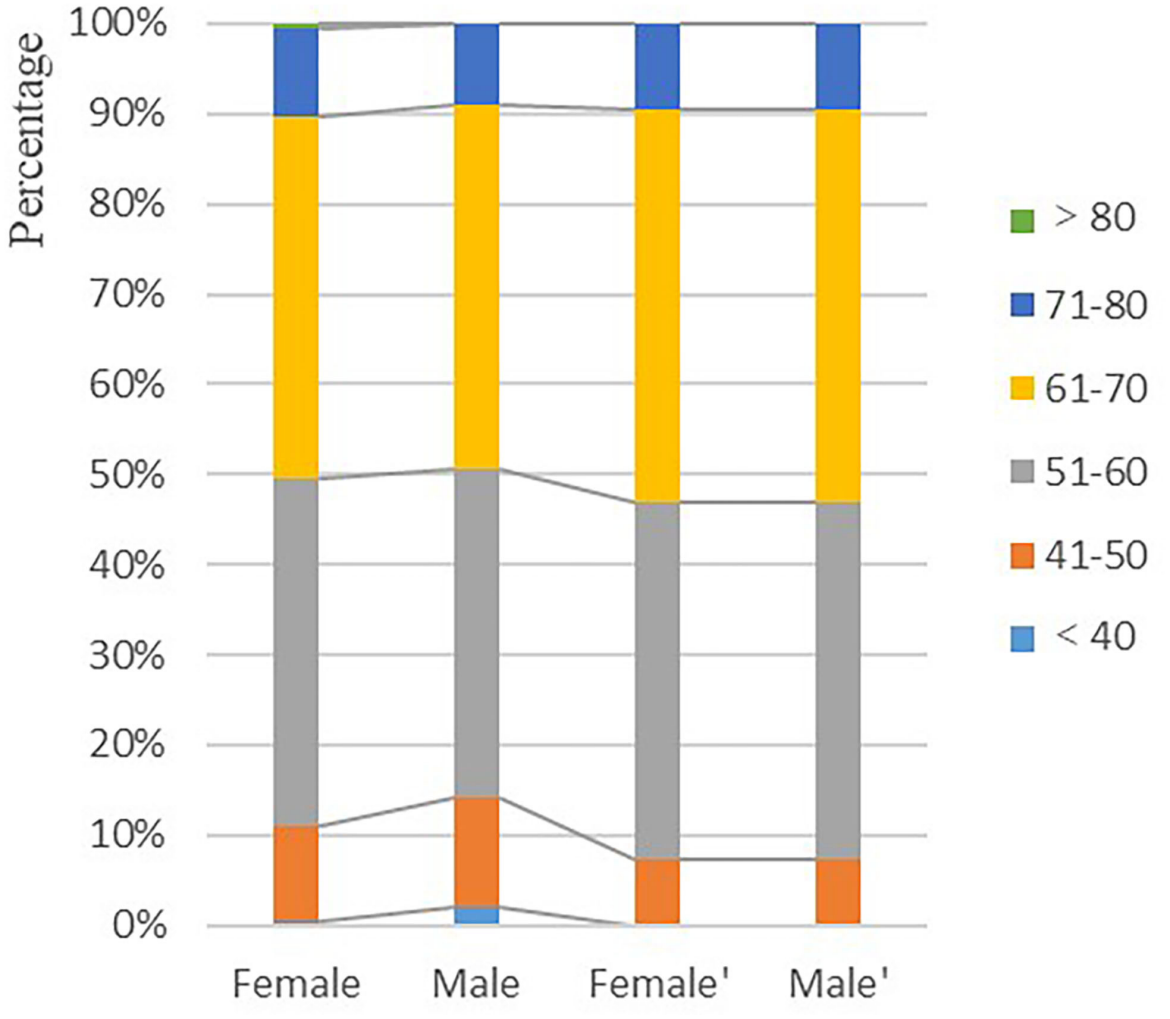
Comparison of the proportion of men and women in different age classes before and after propensity score adjustment. Female and male are pre-adjustment and female′ and male′ are post-adjustment.

In the real world, as well as in previously published articles, intracranial aneurysms are more common in women. Therefore, patients were matched on a 1:2 propensity score based on patient “age class” and “treatment approach,” for male and female patients (Table 2). The mean age of patients was similar in both groups after matching (61.8 ± 7.9 years vs. 60.2 ± 7.4 years, *p* = 0.105), and the proportions were the same for the different age subgroups (*p* = 1; Figure 2). Similarly, the proportion of male and female patients with different treatment approaches was the same after adjustment (*p* = 1). After adjustment, there was still no significant difference in mFisher scores, hydrocephalus, mortality, or intracranial infection between the two groups. Furthermore, the incidence of cerebral ischemia and mRS score ≥2 at discharge remained higher in the female group when compared to the male group.

### Risk Factors of Poor Prognosis

Univariate analysis of patients with poor prognosis (mRS ≥ 2) at discharge showed that age, sex, hypertension, mFisher score, treatment, location, ischemia, and hydrocephalus affected patients' prognosis at discharge. Multivariate analysis including the above factors in a logistic regression analysis revealed high mFisher score (mFisher = 3/4) and ischemia as independent risk factors for poor patient prognosis at discharge (Table 3).

**Table 3:** Multivariate logistic regression analysis of patients with poor prognosis at discharge before propensity adjustment.

**Variables**	**OR (95%CI)**	***P***
Age	1.021 (0.981–1.062)	0.304
Male	0.54 (0.267–1.068)	0.081
Hypertension	1.22 (0.648–2.302)	0.537
**mFisher**						
1	1.444 (0.585–3.585)	0.424
2	1.664 (0.62–4.445)	0.308
3	5.378 (1.979–15.368)	0.001^*^
4	9.322 (3.282–28.907)	<0.001^*^
**Treatment**						
Conservative	3.154 (0.966–10.907)	0.061
Craniotomy	1.157 (0.552–2.434)	0.699
**Location**						
ICA	1.241 (0.503–3.097)	0.64
MCA	2.5 (0.89–7.221)	0.085
PCA	0.673 (0.193–2.292)	0.528
Ischemia	10.261 (3.958–31.182)	<0.001^*^
Hydrocephalus	NA	NA

*ICA, Internal carotid arteries; MCA, Middle cerebral artery; PCA, Posterior circulation artery; NA, Not applicable*.

* *p < 0.05, with a statistical difference*.

Analysis of risk factors for poor prognosis was performed again after propensity score matching. Univariate analysis revealed that sex, mFisher, treatment, and ischemia influenced prognosis at discharge. Multivariate analysis including the above factors in a logistic regression analysis system found that high mFisher score (mFisher = 3/4) and ischemia were independent risk factors for poor patient prognosis at discharge (Table 4).

**Table 4:** Multivariate logistic regression analysis of patients with poor prognosis at discharge after propensity adjustment.

**Variables**	**OR (95% CI)**	***P***
Male	0.625 (0.32–1.196)	0.159
**mFisher**		
1	1.521 (0.63–3.712)	0.352
2	2.374 (0.89–6.474)	0.087
3	6.797 (2.64–18.642)	<0.001^*^
4	9.972 (3.74–29.07)	<0.001^*^
**Treatment**		
Conservative	3.488 (1.02–13.208)	0.052
Craniotomy	1.284 (0.63–2.633)	0.492
Ischemia	9.219 (3.78–26.332)	<0.001^*^
Hydrocephalus	NA	NA

*NA, Not applicable*.

* *p < 0.05, with a statistical difference*.

## Discussion

The main finding of this study was that there were significant sex differences in aSAH outcomes, with female patients having a higher incidence of cerebral ischemia and worse prognosis at discharge. This result is consistent with most previous literature reports. This trend persisted after propensity score adjustment for patient age class and treatment approach. However, risk factor analysis of poor prognosis in patients with aSAH revealed that ischemia and high mFisher score were independent risk factors for poor patient prognosis and not the female sex.

### Sex Differences in aSAH Incidence

Women are more vulnerable to intracranial aneurysms than men, and this difference becomes more pronounced with increasing age, especially after menopause (5, 10), and may be associated with a decrease in estrogen after menopause (11). As there are sex differences in the incidence, more patients with aSAH in clinical practice are females. In addition, studies have also shown that female sex is a risk factor for ruptured intracranial aneurysm (12). Female patients in our study were approximately twice as likely as male patients to have intracranial aneurysm rupture, which is comparable to rates reported in a previous study (12).

There are also sex differences in the characteristics of intracranial aneurysms, with female patients more often having aneurysms originating from the ICA, especially the posterior communicating artery, while male aneurysms are more commonly found in the ACA (13). Women also seem to be more likely than men to have multiple aneurysms (14). In this study, although the proportion of female patients with multiple aneurysms was also higher than that of male patients, the difference was not statistically significant. As in previous studies, however, aneurysms in female patients more commonly originated from the ICA.

### Sex Differences in aSAH Complications

Studies have shown that cerebral ischemia and hydrocephalus are two important complications that affect the prognosis of aSAH (4). As with other studies (15, 16), our study found that the likelihood of cerebral ischemia was significantly higher in female patients with aSAH than in male patients with aSAH. This phenomenon could be because female patients with aSAH are more vulnerable to vasospasm (12). Studies have shown that early vasospasm predicts a higher incidence of SAH-related cerebral ischemia and a poorer prognosis (17). Female sex has been reported to be an independent risk factor for cerebral vasospasm after aSAH (18, 19). Moreover, it has also been found that catecholamine metabolites, which reflect sympathetic excitation, have a higher level in the cerebrospinal fluid of female patients with SAH, which also suggests that they are more vulnerable to vasospasm (20). However, it has also been reported that there are no sex differences in SAH cerebral vasospasm (21). In addition, studies have shown that SAH vasospasm is also associated with inflammatory and genetic factors (22, 23), the characteristic effects of which in women are not well known. Therefore, the reasons and mechanisms for this difference and whether it is associated with estrogen remain uncertain (24, 25).

In addition, some experimental studies have shown sex differences in the occurrence of hydrocephalus after aSAH (26). Some clinical studies have also found female sex to be a risk factor for hydrocephalus after SAH (27). This difference may be related to the ability of estrogen to induce more neutrophils leading to more severe ventricular dilatation and white matter damage (28). However, most studies of sex differences in the prognosis of aSAH did not find that female patients were more likely to develop hydrocephalus (8, 29). In our study, female patients with aSAH were more likely to have hydrocephalus than male patients, but the difference was not statistically significant.

### Sex Differences in aSAH Prognosis

In our study, a higher proportion of female patients had an mRS ≥ 2 at discharge and worse prognosis than the male patients. Although we chose the more stringent criterion of mRS ≥ 2 for poor prognosis, our results are similar to the results of previous studies (30). By contrast, in other studies, they found no significant differences in the prognosis of male and female patients with aSAH (31). They suggested that the difference in age classification between female and male patients may have biased the comparison between the two groups (8). Moreover, different treatment option choices related to surgeon and patient selections may influence the comparison between the two groups (32). However, in our study, the proportion of female patients discharged with an mRS score ≥2 was still significantly higher than that of male patients after adjustment for “age class” and “treatment approach.” Therefore, we suggest that although male and female aSAH patients may have different demographic characteristics and receive treatment using different modalities, the prognosis between sexes is different.

In addition, as in previous studies, our study showed that the occurrence of cerebral ischemia and high mFisher score were independent risk factors for poor patient prognosis (33). As the incidence of cerebral ischemia after aSAH was significantly higher in female patients than in male patients in our study, and there was no significant difference in mFisher score between the two groups, we believe that the poor prognosis of female patients correlates with their higher incidence of cerebral ischemia.

## Limitations

As a retrospective study, our study has inherent limitations. For instance, some information relevant to sex comparisons was not collected (e.g., pregnancies and births), which could suggest changes in estrogen levels in female patients and facilitate the exploration of the role of estrogen in female SAH. Moreover, smoking was not quantified, which might have helped determine the dose-effect relationship between smoking and incidence of aSAH in women (34). In addition, the limited sample size may have prevented us from identifying smaller sex differences, for example, the sex difference in hydrocephalus after aSAH mentioned in the article, which may mean that our results ignore the specific effect of hydrocephalus on the prognosis of female patients.

## Conclusion

Female patients with aSAH have a worse prognosis at discharge compared with that of male patients, and this difference may be because female patients are more vulnerable to cerebral ischemic complications.

## Data Availability Statement

The original contributions presented in the study are included in the article/supplementary material, further inquiries can be directed to the corresponding author/s.

## Ethics Statement

Ethical review and approval was not required for the study on human participants in accordance with the local legislation and institutional requirements. Written informed consent for participation was not required for this study in accordance with the national legislation and the institutional requirements.

## Author Contributions

NX, JC, PJ, and WZ contributed to the study concept and design. YC, JZ, ZLi, TZ, CX, ZLiu, YM, CM, and XC contributed to the acquisition and analysis of data. YC, CJ, JZ, SC, and ZLiu contributed to the image review and drafting figures of the manuscript. NX was responsible for the overall content as guarantor. All authors contributed to the article and approved the submitted version.

## Conflict of Interest

The authors declare that the research was conducted in the absence of any commercial or financial relationships that could be construed as a potential conflict of interest.

## Publisher's Note

All claims expressed in this article are solely those of the authors and do not necessarily represent those of their affiliated organizations, or those of the publisher, the editors and the reviewers. Any product that may be evaluated in this article, or claim that may be made by its manufacturer, is not guaranteed or endorsed by the publisher.
